# From pathogenesis to treatment, a systemic review of cardiac lipoma

**DOI:** 10.1186/s13019-020-01379-6

**Published:** 2021-01-06

**Authors:** Shenglei Shu, Jing Wang, Chuangsheng Zheng

**Affiliations:** 1 Department of Radiology, Union Hospital, Tongji Medical College, Huazhong University of Science and Techonolgy, No.1277 Jiefang Avenue, Wuhan, 430022 Hubei Province China; 2 Hubei Province Key Laboratory of Molecular Imaging, Wuhan, China

**Keywords:** Cardiac lipoma, Noninvasive diagnosis, Clinical management, Systemic review

## Abstract

Cardiac lipoma is an uncommon primary cardiac tumor. With the advancement of diagnostic methods and treatment techniques, more cases of cardiac lipomas have been reported and suggest that the entity previously widely thought to display classic features may also show atypical findings. A systemic review of the rare cardiac tumor was done by searching the literature of cardiac lipoma. We endeavor to summarize the clinical features of the rare disease from pathogenesis to treatment. Literature of cardiac lipoma was retrospectively searched through PubMed and 255 cases of cardiac lipoma were included into this analysis. Cardiac lipomas can occur anywhere within the heart, 53.1% were located within the cardiac chambers, 32.5% in the pericardium, 10,7% within the myocardium and 3.7% involved multiple structures. More than half of the reported cardiac lipomas (66%) may be clinically symptomatic, presenting with symptoms ranging from chest discomfort to syncope depending on their size and location as well as extent of myocardial involvement. Noninvasive cardiac imaging has replaced the role of autopsy and cardiothoracic surgery in detection and diagnosis of cardiac lipomas. Most symptomatic patients (83.7%) were treated by resection of cardiac lipomas and 68.3% of asymptomatic patients also underwentprophylactic resection. Overgrowth and myocardial infiltration of lipomas may result in unsuccessful resection. Recurrence of cardiac lipomas was rare but reported in a few cases. The early detection and accurate diagnosis of cardiac lipoma is of great significance in clinical management, to avoid an unfavourable outcome due to overgrowth.

## Background

Realdo Columbus was credited with the first report of a cardiac tumor in 1559. However, it was not until 1856 that the first cardiac lipoma was reported by Albers. The benign mass is composed of mature adipose tissue and may occur in any site where fat tissue is present. Occurrence of true lipoma within heart and pericardium is rare [1]. By the end of second decade of the twenty-first century, no more than 400 cases of cardiac lipomas had been reported. It’s generally considered that most cardiac lipomas are silent, and symptomatic lipomas can be treated by radical resection [2, 3]. With the advancement of diagnostic tools and treatment techniques, the detection and diagnosis of cardiac lipoma has gained substantial progress and more cases with atypical manifestation of cardiac lipomas have been reported [3, 4]. The aim of this article is to systematically review and analyze the existing knowledge on cardiac lipoma, as well as providing a more comprehensive profile of the rare disease.

### Pathogenesis

Lipomas are soft masses of fat tissue which are often encapsulated by a thin layer of fibrous tissue. Though most cardiac lipomas are composed of mature white adipose tissue, cardiac lipomas composed of fetal brown fat have also been reported in several cases [5–7]. The etiology of the mesenchymal tumor remains unknown. Genetic variation mostly involving the HMGA2 gene is commonly seen in extracardiac lipomas [8]. However, such cytogenetic mutation is uncommon in cardiac lipomas [9]. Multiple cardiac lipomas have been reported in several patients with tuberous sclerosis [10], but their relation is yet to be established.

The natural pathogenetic process of cardiac lipoma is not yet completely understood. On the one hand, they may be symptomatically silent for a prolonged period and even undergo necrobiotic changes [9], whereas in other instances, lipomas may grow to be very large in size with or without infiltration of the myocardium [3, 11, 12]. Pericardial lipomas may even result in myocardium resorption with cavitation and communication with cardiac chambers, thus forming a pseudoaneurysmal appearance [13–16]. In addition, solitary lipomas involving multiple cardiac cavities across the myocardium have been reported [17–19]. If they do not originate from multiple foci, it suggests that the benign mass has great capacity of infiltrative growth. The infiltrative growth pattern has been postulated as the result of gradual invagination of the firm lipomas into the pliable cavity wall within the process of repetitive systolic contraction [20]. No evidence is available that cardiac lipomas may undergo malignant transformation, but mature lipomas and well-differentiated liposarcomas may coexist within one heart [21].

### Epidemiology

True lipomas with fibrous encapsulation occurring in heart and pericardium are very rare. The reported incidence of primary heart tumors in the autopsy series is between 0.2–0.4% [22, 23], of which cardiac lipomas account for 8.4% [10]. As these figures include both highly encapsulated lipomas and lipomatous hypertrophy of interatrial septum, the exact incidence of cardiac lipoma may be overestimated. As for lipomas composed of fetal brown adipose tissue, the so called hibernoma, these are extremely rare in the heart and only few cases have been reported [5–7]. By searching PubMed, we found that more than 300 cases of true cardiac lipomas have been reported. Two hundred fifty-five cases of cardiac lipoma with available details were included and reviewed.

**Fig. 1:**
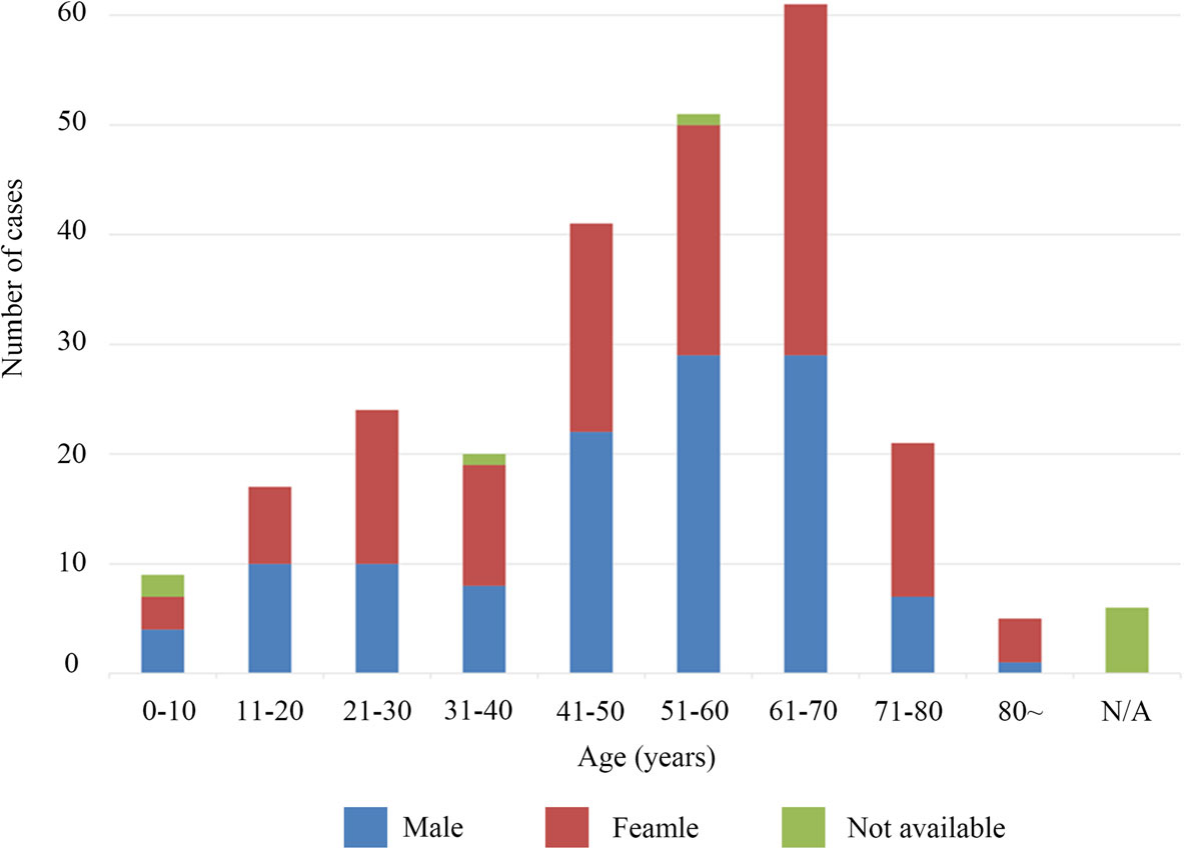
Age and sex distribution of cardiac lipomas of 255 reported cases

Among the included cases, no difference in the distribution of cardiac lipoma between genders (126 female, 120 male, in 9 cases gender is not available) was shown. Cardiac lipomas may occur at any age of life from the fetal period to the elderly in their 80s [24, 25]. Most cardiac lipomas occur in the 40–70 age group (153/255, 60.0%, Fig. 1). Among the included reports, 242 patients (242/255, 94.9%) had single cardiac lipoma, 13 patients (13/255, 5.1%) had multiple (2 or more) cardiac lipomas. Of note, 5 encapsulated cardiac masses in 4 patients were composed of brown adipose tissue, thus constituting a diagnosis of cardiac hibernoma.

### Characteristics

The different sites of origin of lipomas within the heart have not been described in detail. The commonly described locations of origin are: 25% from the subpericardium, 25% from the myocardium and 50% from the subendocardium [2, 26, 27]. As the mechanism of cardiac lipoma formation is not yet fully understood, the origin of cardiac lipoma is determined by the location and attachment.

**Fig. 2:**
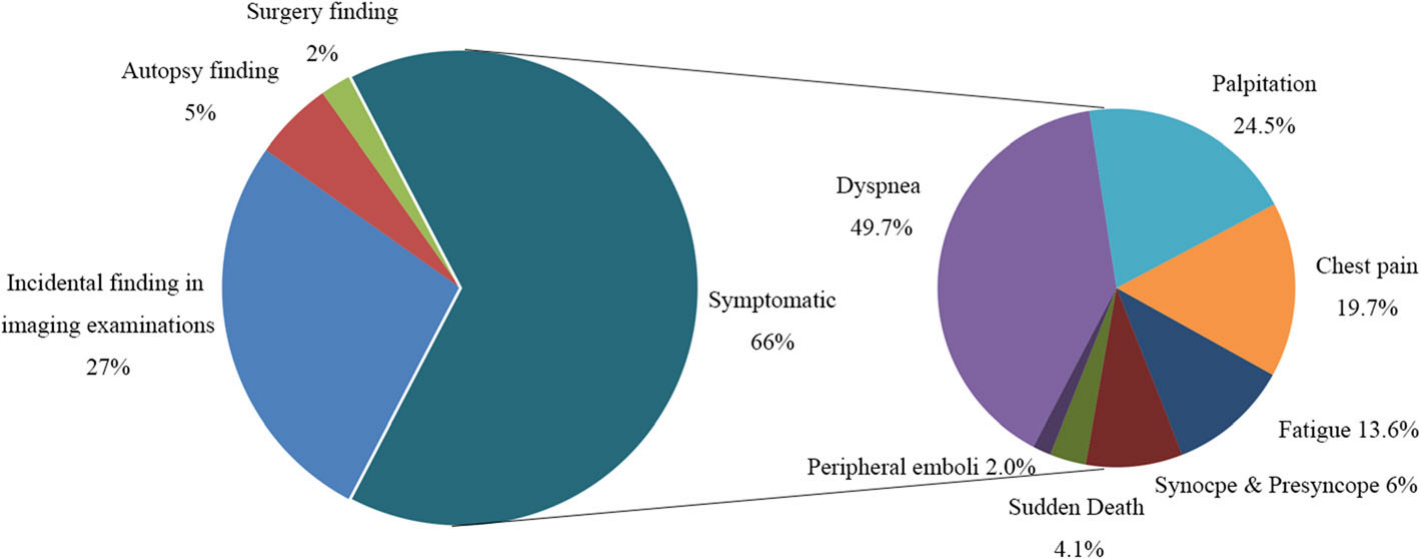
Site distribution of cardiac lipomas across heart and pericardium of 255 reported cases. LV: left ventricle; RA: right atrium; RV: right ventricle; LA: left atrium

By reviewing the location of cardiac lipomas in the included reports, there were more than 271 cardiac lipomas in 255 patients. 144 (144/271, 53.1%) lipomas were located within the cardiac chambers, of which 61 (61/144, 42.4%) were in the right atrium, 48 (48/144, 33.3%) in the left ventricle, 19 (19/144, 13.2%) in the right ventricle, and 4 (4/144, 2.8%) in the left atrium (Fig. 2). In particular, 12 (12/144, 8.3%) originated from cardiac valvular leaflets. 88 (88/271, 32.5%) lipomas were found in the pericardium. 29 (29/271, 10.7%) lipomas were located within the myocardium of ventricular or atrial wall. Although lipomatous hyperplasia is commonly seen in the atrial septum, most intramyocardial lipomas occurred within the left ventricular wall (11 in interventricular septum, 8 within free wall of left ventricle). The other 9 (9/271, 3.3%) lipomas involved multiple chambers. One special lipoma (1/271, 0.4%) was found as an embolus occluding the left coronary ostium in autopsy of a patient with sudden death [28]. Their distribution within the heart suggests that around 48.7% lipomas arise from the subendomyocardium (132/271), 32.5% (88/271) from subpericardium, 10.7% (29/271) from myocardium and 4.4% (12/271) from cardiac valvular leaflets. However, this may be an inaccurate analysis, as myocardial infiltration of intracardiac or intrapericardial masses may falsely suggest that the lipoma arise from the myocardium. Furthermore, the original site of a solitary lipoma involving multiple cavities across the myocardium cannot be easily determined. With more cases to be reported, the distribution of cardiac lipomas may be more accurately described in the future.

### Clinical presentation

**Fig. 3:**
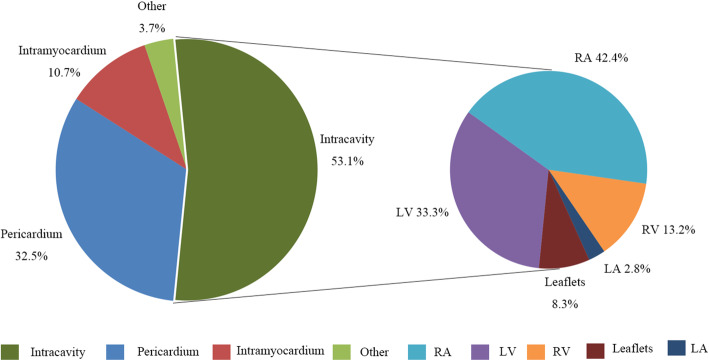
Clinical manifestations of 255 patients with cardiac lipomas

It is generally assumed that most cardiac lipomas are silent and only a small proportion of this entity may show clinical symptoms depending on their location and size [3]. However, among the included cases, only in 97 patients (97/255, 38.0%) were cardiac lipomas found incidentally without related symptoms. Among them, 76 cases of lipomas were detected on routine check-up or examination for other purposes (76/255, 29.8%), 15 cases of lipomas were detected on autopsy in patients who died from other causes (15/255, 5.9%), and the remaining 6 patients’ lipomas were discovered incidentally during surgery for other purposes (6/255, 2.4%). Symptoms related to cardiac lipoma were presented in 147 patients (147/255, 57.6%) ranging from mild chest discomfort to sudden death. Dyspnea is the most common symptom and this was reported in 73 patients (73/147, 49.7%). Palpitations (36/147, 24.5%), chest pain (29/147, 19.7%) and fatigue (20/147, 13.6%) are also common complaints of symptomatic patients. Syncope or presyncope have been reported in 16 (16/147, 10.9%) patients, and this was due to severe arrhythmia or obstruction of ventricular outflow tract. Six patients had sudden death due to severe arrhythmia or acute coronary occlusion associated with cardiac lipoma. Very rarely, peripheral embolic events presented as initial symptoms in 3 patients with cardiac lipoma [29–31], which were believed to be caused by the detached thrombi related to arrhythmia (Fig. 3).

**Fig. 4:**
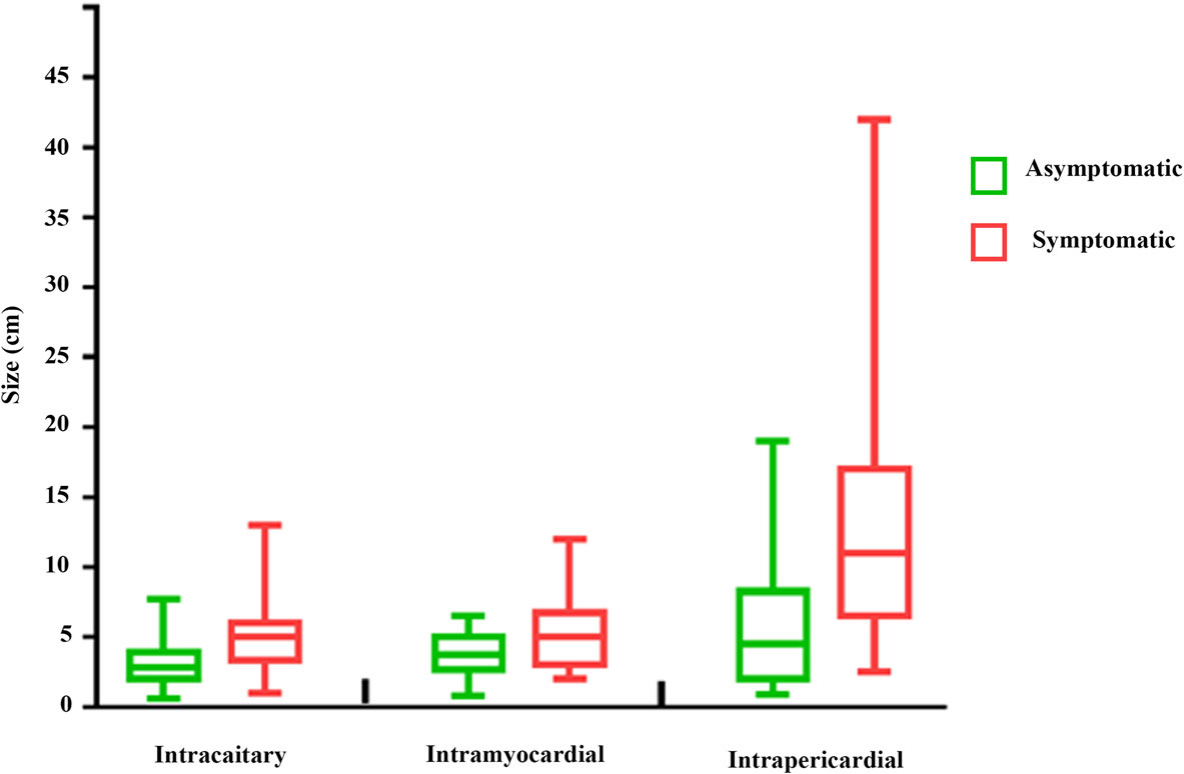
Comparison of size between intracardiac, intramyocardial and intrapericardial lipomas

Cardiac lipomas at different sites may cause clinical presentation by different pathways. For lipomas within cardiac cavities, obstruction of blood flow may cause symptoms varying from fatigue to even syncope [32–34]. Large pericardial lipomas can be symptomatic by compressing cardiac chambers, vessels or coronary arteries [21, 35, 36]. Myocaridal lipomas are prone to cause conduction abnormalities by compression or infiltration of the conduction system [37, 38]. The size of the cardiac lipoma varied with distribution. In general, pericardial lipomas may grow extensively within the pericardial sac and shared larger size (median, 10.0 cm) than intracavitary (3.5 cm) and myocardial lipomas (4.0 cm). Their size is also closely related to the clinical presentation. Median size of symptomatic lipomas is much larger than those found incidentally (6.1 cm vs 3.0 cm) (Fig. 4). However, small lipomas at special sites such as cardiac valves may also cause severe symptoms [39, 40].

### Diagnosis

Historically, cardiac lipomas were incidental findings during autopsy and cardiothoracic surgery. Noninvasive detection of cardiac masses including lipomas in vivo was not possible until the clinical application of X-ray imaging methods. Lipomas within cardiac cavities may be detected as intracavitary filling defects in cardioangiography [41, 42], while lipomas within pericardial space may show nonspecific signs of an enlarged heart on chest radiograph resembling pericardial effusion [43, 44]. Application of echocardiography made the detection of cardiac masses including lipoma a big step forward. Echocardiography has incomparable advantages of ready availability, convenient operation and radiation-free and it remains the preferred screening method for cardiac masses. Cardiac lipomas usually present as homogenous hyperechoic masses within cardiac chambers or hypoechoic masses within the pericardium [45, 46]. Although most cardiac lipomas can be sensitively detected and accurately located by echocardiography, their nature cannot be determined based on acoustic property [47]. Acoustic characteristics of lipomas may help exclude cardiac malignancies, and differentiation with other benign lesions such as myxoma is difficult [3].

**Fig. 5:**
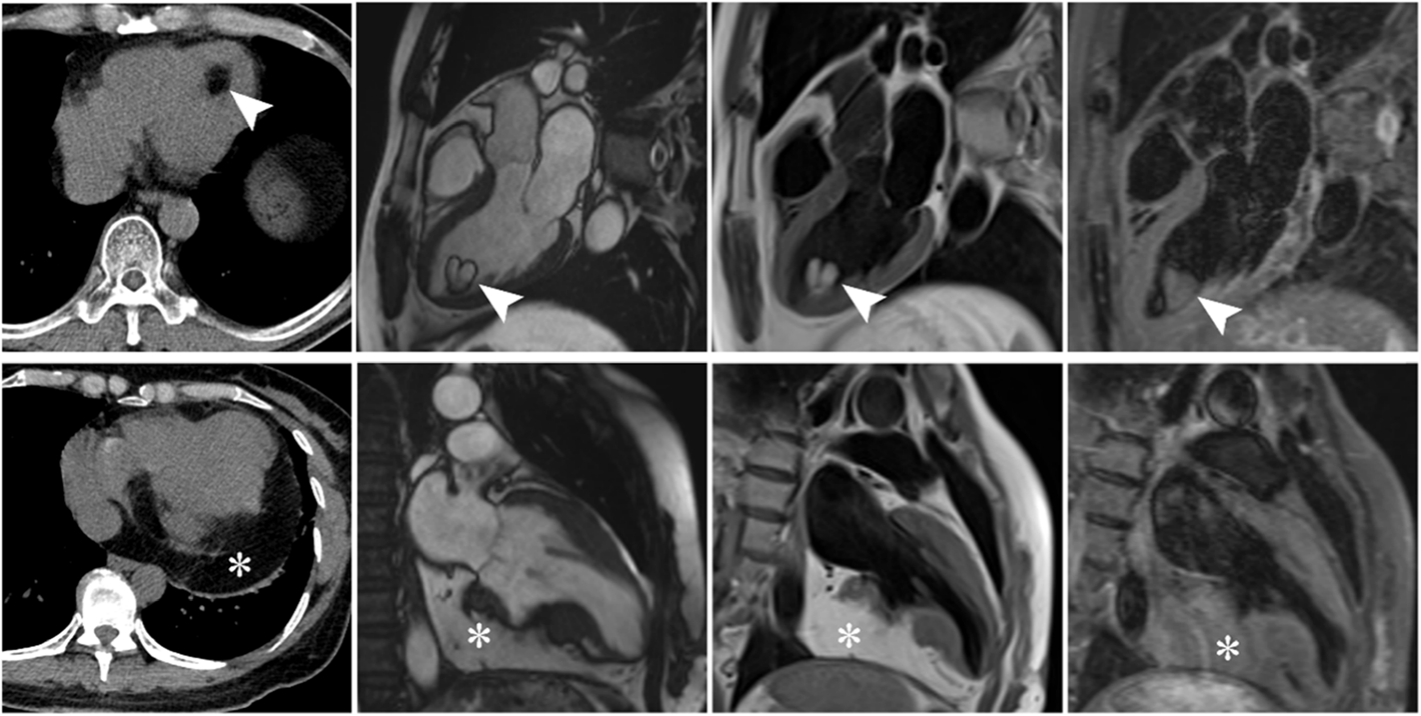
CT and MR images of left ventricular (triangular arrows) and pericardial (asterisk) lipomas. Imaging features of cardiac lipomas show consistence with subcutaneous fat in all sequences

Emergence of cross-sectional imaging methods including computed tomography (CT) and magnetic resonance imaging (MRI), especially the latter one, made noninvasive diagnosis of cardiac lipoma possible [48, 49]. Lipomas have entirely the same composition with mature adipose tissue, they show the same imaging appearances with subcutaneous fat in CT and MRI on all sequences [50, 51] (Fig. 5). On CT, they present as homogenous hypodense encapsulated masses with or without linear septa (Hounsfield measurement <− 50) [52]. Signal features of cardiac lipomas show consistence with subcutaneous fat in all MR sequences. Specifically, the complete signal loss of the mass on fat suppression sequence is characteristic for the diagnosis of lipoma [53, 54]. Of note, the black boundary sign in cine sequence due to chemical shift effect is of great help in diagnosing small lipomas [55]. Recently, the tissue mapping technique applied in cardiac imaging has shown great potential to quantitatively diagnose cardiac lipoma [55]. Hemodynamic state, which is commonly evaluated by echocardiography, can also be assessed in cardiac MRI real-time cine sequence. Over all, myocardial infiltration of lipoma can be sensitively detected with cardiac MRI [56]. Like lipoma elsewhere, diagnosis of cardiac lipomas can be established with plain scan, and they do not demonstrate any enhancement on contrast scanning [57]. Though imaging features of cardiac lipoma are highly specific on CT and MRI, it is occasionally necessary to differentiate this benign entity from liposarcoma. The presence of nonuniformly thick septa and mass-like nonadipose areas may suggest the diagnosis of liposarcoma [58]. The differentiation between mature cardiac lipomas and well-differentiated liposarcoma may occasionally be difficult by imaging [21]. In summary, echocardiography plays an irreplaceable role in screening of cardiac lipoma, while CT and MRI (particularly MRI), is indispensable for accurate diagnosis and comprehensive evaluation.

### Treatment

There is no guideline on the treatment of cardiac lipoma. Since the first successful removal of a pericardial lipoma reported in 1952 [59], more and more symptomatic cardiac lipomas have been resected, especially after the introduction of the cardiopulmonary bypass technique into cardiac surgery. Radical resection was adopted to relieve symptoms caused by cardiac lipomas in most patients [60, 61]. Among the included reports, most symptomatic patients (83.7%, 123/147) underwent resection of cardiac lipomas. Although conservative management may be implemented in patients without symptoms related to cardiac lipoma, 56 patients with (68.3%, 56/82) asymptomatic cardiac lipomas also received prophylactic resection. Recurrence of cardiac lipomas after surgical resection is extremely rare but has been reported in few cases [4, 12]. In such cases, incomplete removal due to diffuse infiltration in the myocardium seems to be the contributing factor. Resection of the recurrent lipoma was extremely challenging and heart transplantation may provide the ultimate solution. Over all, radical resection should be considered in all patients with cardiac lipoma, as asymptomatic lipomas may undergo overgrowth and infiltration into the myocardium, and this may result in unfavorable outcomes upon resection. Additionally, close follow-up with imaging methods should be provided toall patients to monitor lipoma overgrowth or recurrence [62, 63].

## Conclusion

Cardiac lipoma is a benign primary tumor of the heart. Nonetheless, it may present with clinical symptoms varying from mild discomfort to syncope. Overgrowth of lipoma and infiltration into the myocardium may indicate a more severe clinical presentation and unfavorable outcome. Accurate diagnosis and comprehensive evaluation of cardiac lipoma is highly dependent on multimodality imaging methods. Radical resection of the lipoma is the optimal method of treatment in symptomatic patients. Conservative management may be implemented for asymptomatic cardiac lipomas and prophylactic resection should also be considered.

## Data Availability

Not applicable.
